# EBV-induced T-cell responses in EBV-specific and nonspecific cancers

**DOI:** 10.3389/fimmu.2023.1250946

**Published:** 2023-09-29

**Authors:** Qiuting Zhang, Miao Xu

**Affiliations:** State Key Laboratory of Oncology in South China, Guangdong Key Laboratory of Nasopharyngeal Carcinoma Diagnosis and Therapy, Sun Yat-sen University Cancer Center (SYSUCC), Guangzhou, Guangdong, China

**Keywords:** Epstein-Barr virus, T-cell immunity, EBV-associated cancer, EBV-specific T cells, CAR-T, γδ T-cells, tumor-associated antigens (TAAs)

## Abstract

Epstein-Barr virus (EBV) is a ubiquitous human tumor virus associated with various malignancies, including B-lymphoma, NK and T-lymphoma, and epithelial carcinoma. It infects B lymphocytes and epithelial cells within the oropharynx and establishes persistent infection in memory B cells. With a balanced virus-host interaction, most individuals carry EBV asymptomatically because of the lifelong surveillance by T cell immunity against EBV. A stable anti-EBV T cell repertoire is maintained in memory at high frequency in the blood throughout persistent EBV infection. Patients with impaired T cell immunity are more likely to develop life-threatening lymphoproliferative disorders, highlighting the critical role of T cells in achieving the EBV-host balance. Recent studies reveal that the EBV protein, LMP1, triggers robust T-cell responses against multiple tumor-associated antigens (TAAs) in B cells. Additionally, EBV-specific T cells have been identified in EBV-unrelated cancers, raising questions about their role in antitumor immunity. Herein, we summarize T-cell responses in EBV-related cancers, considering latency patterns, host immune status, and factors like human leukocyte antigen (HLA) susceptibility, which may affect immune outcomes. We discuss EBV-induced TAA-specific T cell responses and explore the potential roles of EBV-specific T cell subsets in tumor microenvironments. We also describe T-cell immunotherapy strategies that harness EBV antigens, ranging from EBV-specific T cells to T cell receptor-engineered T cells. Lastly, we discuss the involvement of γδ T-cells in EBV infection and associated diseases, aiming to elucidate the comprehensive interplay between EBV and T-cell immunity.

## 1 Introduction

Epstein-Barr virus (EBV), also known as human herpesvirus 4 (HHV-4), is a highly prevalent γ-herpesvirus that infects an overwhelming 90% of the adult population worldwide (1). Since its discovery in 1964 from a Burkitt lymphoma cell line, extensive research has been conducted to investigate its association with cancer (2). In 2020, EBV-associated cancers accounted for an estimated 239,700 to 357,900 new cases and caused 137,900 to 208,700 deaths globally (3). EBV is considered the primary etiological agent associated with multiple epithelial and lymphoid cancers of variable fractions, including nasopharyngeal carcinoma (NPC), gastric carcinoma (GC), Hodgkin lymphoma (HL), Burkitt lymphoma (BL), Diffuse large B-cell lymphoma (DLBCL) and Extranodal NK/T-cell lymphoma, Nasal type (ENKTL-NT). In addition, EBV reactivation can lead to uncontrolled B-cell proliferation in immunocompromised individuals, including post-transplant lymphoproliferative disease (PTLD) in hematopoietic stem cell transplant (HSCT) or solid organ transplant (SOT) recipients and B-cell lymphoma in AIDS patients (4–6).

Despite its ubiquity, most people remain asymptomatic throughout their lifetime, owing to the potent host immune system, especially its cellular immunity, which keeps the virus at bay. However, when cellular immunity is compromised or dysregulated, the virus can replicate unchecked, leading to EBV-associated B-cell malignancies (7). These malignancies express EBV antigens that T cells can specifically target (8). Over the last two decades, the encouraging outcomes of adoptive cell therapy using EBV-specific T cells in treating PTLD have sparked significant research interest. Many clinical trials have been launched to explore their potential application in treating other EBV-related malignancies (9). Recent studies find that EBV latent membrane protein 1 (LMP1), upon ectopic expression in EBV-unrelated cancers, can upregulate TAAs and induce a robust TAA-specific CD4+ CTL response (10), indicating that beyond its oncogenic implications, EBV also has the potential for therapeutic applications in cancer treatment. This review aims to advance our understanding of the roles of T-cell immunity across both EBV-related and unrelated cancers and provide insights to devise more effective immune-based cancer prevention and treatment strategies.

## 2 Biology of EBV and EBV-associated cancers

The transmission of EBV occurs through oral means and involves the infection of epithelial cells of the oropharynx, followed by replication and spread to B cells, which are major sites for EBV infection in humans. While EBV predominantly targets B lymphocytes and epithelial cells, it can sporadically infect other human cell types, including T cells and natural killer cells, albeit infrequently (11–13). EBV life cycle is complex and is composed of latent and lytic infections. Only nine proteins contributing to B cell transformation and tumorigenesis are expressed during latent infection. These include six EBV nuclear antigens (EBNA-1, -2, -3A, -3B, -3C, and -LP) and three latent membrane proteins (LMP-1, -2A, and -2B). The latent cycle can be subdivided into four patterns, namely latency III, II, I, and 0, characterized by gradually restricted viral gene expression patterns to evade immune surveillance. Ultimately, EBV establishes persistent residence in memory B cells, characterized by the absence of viral antigen expression (latency 0), thereby evading T-cell recognition and acting as a viral reservoir. The latent-lytic switch is a particularly significant event in the EBV life cycle, but its mechanism remains elusive. EBV can transition to the lytic cycle periodically, resulting in viral replication, shedding, and subsequent transmission (8, 11, 12, 14).

During lytic infection, EBV expresses more than 80 lytic proteins that facilitate the generation of new viral particles (8). The viral lytic cycle is divided into three temporal and functional stages: immediate early (IE), early (E), and late (L). IE gene products are transcription factors in charge of turning on the cascade of expression of lytic genes. Among these proteins, the immediate early proteins BZLF-1 and BRLF-1 act as triggers of the EBV lytic cycle (15). E genes encode enzymes with DNA replication function, and L genes are mostly viral structural proteins.

Several lytic genes are somewhat expressed during latent states. For instance, BHRF1, commonly associated with the virus lytic cycle, remains constitutively expressed as a latent protein *in vitro* within growth-transformed cells and might contribute to virus-associated lymphomagenesis in Wp-restricted BL (16). Additionally, BALF1, expressed with early kinetics during the lytic cycle, is found in latently infected epithelial and B cells (15). While dispensable for lytic replication and B cell transformation, BALF1 might facilitate efficient transformation, potentially *in vivo* (15).

Under specific circumstances (17), such as immunosuppression like HIV or immunosuppressive therapy (18), concurrent infections such as CMV, HPV, or coronavirus (19, 20), disruptions in cellular equilibrium like hypoxia (21), or psychological stressors like familial and socio-economic instability (22), EBV can switch from latency to lytic infection, termed viral reactivation, contributing to the dissemination of the virus and its potential to cause various diseases and complications.

**Figure 1:**
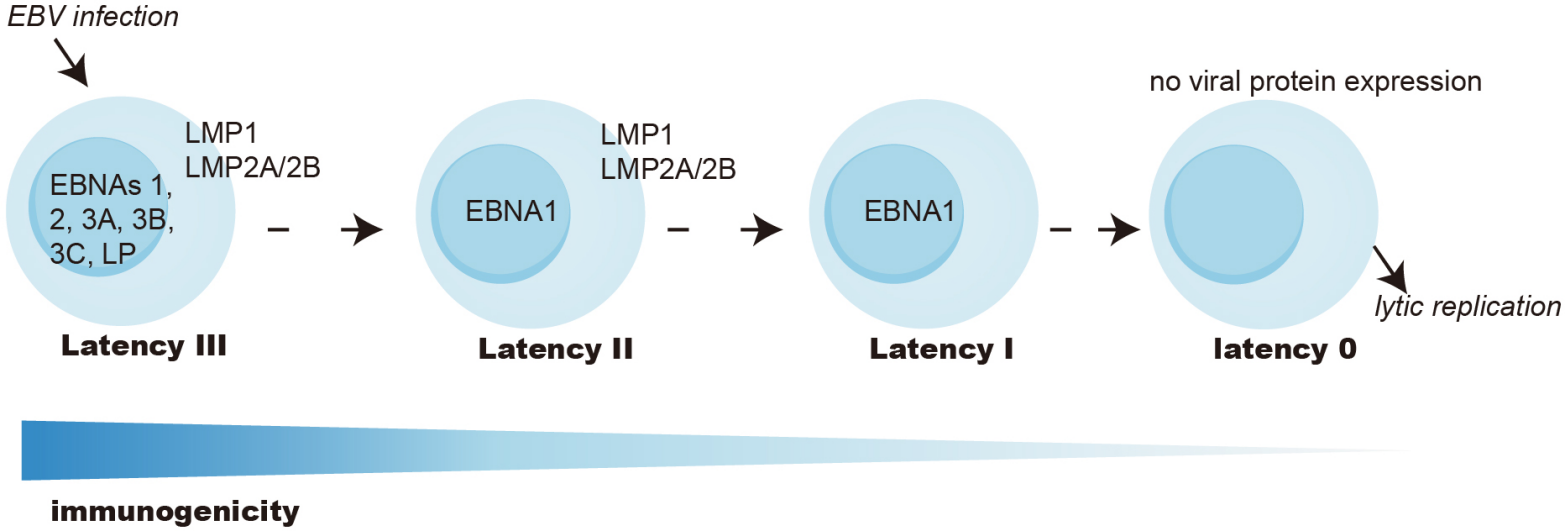
EBV latency types. Latency III express all EBV-encoded latent proteins and is the most immunogenic. Latency II expresses EBNA1, LMP1, and LMP2, and has intermediate immunogenicity. Latency I only expresses EBNA1 and is poorly immunogenic. Latency 0 abolishes all antigen expression and is seen in memory B cells as a reservoir of the virus. There can be transition latency states with upregulation of latency and lytic genes. EBNA, EBV nuclear antigen; LP, leader protein; LMP, latent membrane protein.

In EBV-associated cancers, latent EBV proteins are crucial for tumor pathogenesis, and their expression can classify tumors into distinct categories (**Figure 1**). In type III latency cancers, cells infected with EBV express a full array of latent proteins, including six EBV nuclear antigens (EBNA1, 2, 3A, 3B, 3C, LP), two latent membrane proteins (LMP1, 2), BamH1-A right frame 1 (BARF1), several small noncoding RNAs, various micro-RNAs, and EBV-encoded small RNAs. All EBNA3 family proteins are highly immunogenic and can be effectively targeted and cleared by T cells in immunocompetent individuals (8, 23, 24). Consequently, type III latency malignancies can primarily be seen in innate or acquired immunodeficient individuals, such as PTLD of HSCT or SOT recipients and B-cell lymphoma in AIDS patients. Type III latency can also be seen in EBV-transformed B cell lymphoblastoid cell lines (LCLs) cultured *in vitro*.

Type II latency tumors mainly include NPC, GC, some cases of HL, and NKT. These tumors express EBNA1, LMP1, LMP2, and BARF1 and have intermediate immunogenicity.

Type I latency, marked by sole EBNA1 expression, is seen in BL and exhibits constrained immunogenicity.

Apart from latent antigens, some lytic cycle transcripts are also found in certain tumors, which encode molecules known to contribute to tumor growth (25). Among these transcripts, BZLF1 and BRLF1 are the IE transcription factors that master-regulate EBV reactivation/lytic expression. Notably, the expression of some of the immediate early genes, such as BZLF1, in the absence of other lytic genes, particularly those encoding late structural proteins, thereby precluding the formation of infectious viral particles, is termed the abortive lytic cycle. Specifically, it is known as the pre-latent abortive lytic cycle when it occurs just after infection. The abortive lytic cycle has been well-documented in pre-latent cells (26–30) and established tumors (31–34). Furthermore, evidence derived from mouse models (35, 36) supports the notion that the abortive lytic cycle facilitates cell-to-cell viral dissemination and contributes to viral-induced tumorigenesis.

## 3 EBV-specific T cell immunity in EBV-related cancers

### 3.1 EBV-positive lymphoma in immune-deficient host

In the context of immunocompromised SOT or HSCT recipients, PTLD predominantly arises, characterized by the presence of six EBNA and two LMP antigens denoting Type III latency. The EBNA3 antigens within PTLD demonstrate notable immunogenicity, forming a foundation for potential adoptive cell therapies targeting these specific antigens.

Front-line therapies for PTLD post-HSCT or SOT commonly involve reducing immunosuppression, often coupled with rituximab and occasionally augmented by chemotherapy. However, cellular therapy remains the primary option in cases of inadequate response or relapse. The rich diversity of EBV antigens expressed in these tumors facilitates the efficacy of adoptive therapy using virus-specific cytotoxic T cells (CTLs). Clinical trials across global centers have successfully employed CTL preparations, sourced either autologously or from third-party donors, for PTLD treatment or prevention, with a strong record of safety and efficacy. These antigen-specific T cells are primed via *in vitro* exposure to LCLs. The potent immunogenicity of the EBNA3 family proteins makes them the principal targets of CD8 T-cell immunity (8, 23, 24). CD4+ T cells, though less frequent, also contribute to tumor control (10, 37, 38). CD4+ T-cell effectors are crucial in limiting early-stage EBV-induced B-cell proliferation, and some direct target EBV-transformed LCLs (37). Notably, EBV-specific T cell products enriched with CD4+ T cells correlate with improved clinical outcomes (38). Furthermore, the expansion of T cells through LCL generates CD4+ T cells specific to nonviral cellular antigens (39, 40), known as TAAs (10), upregulated by LMP1 in EBV-infected cells.

### 3.2 EBV-positive tumors in the immunocompetent host

Unlike PTLD, which expresses a full array of EBV latent antigens (latency III), most EBV-associated cancers exhibit limited expression of EBV latent antigens in relatively immunocompetent hosts (**Figure 1**). Immunodominant proteins such as EBNA2, 3A, 3B, 3C, and -LP are absent, redirecting immune attention towards remaining target antigens, such as EBNA1 in BL, EBNA1, LMP1, and LMP2 in HL, and primarily EBNA1 and LMP2 in NPC, GCa, ENKTL, and DLBCL. Efficient recognition of these EBV antigens by T cells is crucial for targeting and eliminating infected cells.

Traditionally considered immunologically inert, EBNA1 has a glycine-alanine repeat (GAr) region that shields it from proteasome breakdown and MHC I presentation (41). However, studies of CD8+ T cells targeting specific EBNA1 epitopes are also reported (42, 43). These T cells can recognize naturally expressed native EBNA1 protein within EBV-transformed LCLs, inhibiting LCL proliferation (44), suggesting that the GAr domain within EBNA1 does not confer complete protection from MHC class I presentation. *In vitro* models suggest that HL, NPC, and T/NKL cells retain MHC class I antigen processing capabilities and can be recognized by CD8+ T cells specific to LMP2 (45–49).

In contrast, BL is deficient in MHC class I processing (50) but exhibits MHC class II expression (51), allowing recognition by EBNA1-specific CD4+ T cells ex vivo and in murine models (52, 53). Besides MHC molecules, HLA polymorphism, which influences antigen presentation and immune recognition, is strongly associated with disease risk (54–57). For example, the HLA-A01 allele increases the risk of EBV-positive HL, whereas HLA-A02 has a protective effect (58). Despite EBV-specific T cells being restricted by various HLA alleles, the emergence of EBV-positive tumors cannot be solely attributed to antigen-specific blindness in the T cell repertoire. T-cell population deficiencies and attenuated T-cell responses are plausible contributors (59, 60). This is particularly evident in endemic BL, where Plasmodium falciparum and EBV act as co-factors in cancer development (61). Malaria stimulates the proliferation of latently infected B cells through viral reactivation (53). Meanwhile, T-cell control of EBV-infected B cells is lost during P. falciparum malaria (59, 60), possibly contributing to an increased risk of incidence of BL.

Furthermore, EBV-positive cancers employ diverse strategies to evade immune surveillance. The tumor microenvironment (TME) within EBV-associated malignancies, including HL, NPC, and the majority of EBV-positive gastric cancers, is characterized by an “immune hot” phenotype (58, 62, 63). These tumors display pronounced infiltration of lymphocytes whose specificities and functions remain incompletely elucidated.

EBV-positive HL exhibits distinct characteristics compared to EBV-negative HL. Notably, the signature of EBV+ cHL tissues is enriched in genes characteristic of Th1 and antiviral responses. Furthermore, in pediatric cases of EBV+ cHL, a robust T cell infiltration is evident, exhibiting a cytotoxic/Th1 immune profile (64, 65). However, markers of suppression also increase, including LAG-3 and IL-10 (66). Regulatory T cells (Tregs), both natural and induced, are present in higher frequencies, contributing to immunosuppression (66, 67). EBNA1 may upregulate CCL20 expression, promoting the migration and recruitment of Tregs (68). Additionally, active signaling by LMP1 and LMP2 can induce high-level expression of galectin-1 and PD-L1 (69–71).

Undifferentiated NPC is invariably EBV-positive and exhibits a suppressive TME characterized by dysfunctional lymphocyte infiltration. Regulatory CD4+ T cells are elevated in the blood and consistently detected in tumors (72). CD8+ FoxP3+ lymphocytes with suppressive functions are also present (73). Immune checkpoint molecules such as PD-L1, LAG3, galectin 9-TIM3, TIGIT, and CTLA4 are overexpressed (74–77). Recently, an epithelial-immune dual feature of NPC cells has been identified, characterized by upregulated MHC II gene expression. This dual feature correlates with CD8+ T cell exhaustion and a suppressed TME, ultimately associated with poor prognosis (78).

Approximately 10% of gastric cancers are EBV-positive (79), and patients with EBV-associated gastric cancer (EBVaGC) have a more favorable prognosis compared to their EBV-negative counterparts (80). EBV-positive gastric cancer exhibits pronounced lymphocytic infiltration (81). Many perforin-positive CD8+ T cells are observed within this infiltrate, exhibiting effectiveness in eliminating autologous EBV-transformed cells (82). However, these CD8+ T cells may not recognize known EBV latent antigenic peptides, suggesting the involvement of alternative cellular antigens (82). Nevertheless, these CD8+ T cells can be counteracted by localized immunosuppression, as evidenced by high expression of PD-L1, PD-L2, and indoleamine 2,3-dioxygenase (IDO), which inhibits T and NK cell function through tryptophan depletion (83, 84).

Despite the diverse repertoire of immunomodulatory mechanisms employed by EBV-positive cancers, adoptive transfer of EBV-specific T cells has demonstrated clinical efficacy in patients with PTLD, HL, NPC, and T/NKL (85–88). The therapeutic effect of EBV-specific T cells not only destroys tumor cells and reduces tumor burden but may also induce the release of potentially antigenic debris from tumor cells, thereby stimulating an immune response against nonviral cellular antigens. This phenomenon, known as epitope spreading (85), expands the range of targeted antigens for T-cell recognition and response. However, the origin of these cellular antigens, whether from epitope spreading or as a consequence of LMP1 signaling-induced upregulation of TAAs on B cells (10), warrants further investigation.

### 3.3 HLA susceptibility

The human leukocyte antigen (HLA) complex, located within the major histocompatibility complex (MHC) on chromosome 6p21.3, plays a vital role in antigen presentation to the immune system. The MHC region encompasses three subregions: HLA class I, crucial for CD8+ T-cell cytotoxicity induction; HLA class II, involved in CD4+ helper T-cell responses; and class III, housing non-HLA genes associated with inflammation, leukocyte maturation, and the complement cascade.

HLA’s diversity and polymorphism contribute to its ability to recognize and target various pathogens. Growing evidence suggests that HLA variations can influence genetic susceptibility to EBV-associated cancers. Notably, NPC strongly associates with HLA genes in the MHC region (54–57). In the genomic analysis of NPC patients, a notable frequency of aberrations in MHC class I genes (NLRC5, HLA-A, HLA-B, HLA-C, B2M) has been observed (89). An HLA class I region-specific association suggests the importance of CD8+ T-cell cytotoxicity in NPC etiology (90). HLA associations may vary across racial groups, with specific HLA alleles conferring protective or increased risk effects in different populations. In Southern China and Southeast Asia, where NPC is most prevalent, HLA-A11 and B13 are associated with a protective effect against NPC, whereas HLA-A02 (A0207, A0206), A33, B46, and B58 are linked to an increased risk of NPC (91).

HLA also demonstrates significant links with other EBV-associated cancers, including HL, BL (92), and PTLD (93). For example, the HLA-A01 allele increases the risk of EBV-positive HL, whereas HLA-A02 has a protective effect (58). However, the mechanisms underlying the diverse roles of HLA alleles in cancer susceptibility and immune escape remain incompletely understood.

In addition to classic HLA genes, non-classic HLA genes have been implicated in immune escape. HLA-G, known to inhibit T-cell and NK-cell function, is frequently expressed in NPC tumors and is associated with poor survival outcomes (94).

Due to its strong association with cancer etiology, HLA has potential applications in cancer screening, as demonstrated in improved prediction efficiency for NPC screening when combining HLA class I gene variants with EBV genetic variants and epidemiological risk factors (95).

To advance our understanding of the intricate role of HLA genes and their interplay with T-cell immunity in EBV-associated cancers, larger-scale and comprehensive studies are needed.

## 4 EBV-induced T cell responses against TAAs

**Figure 2:**
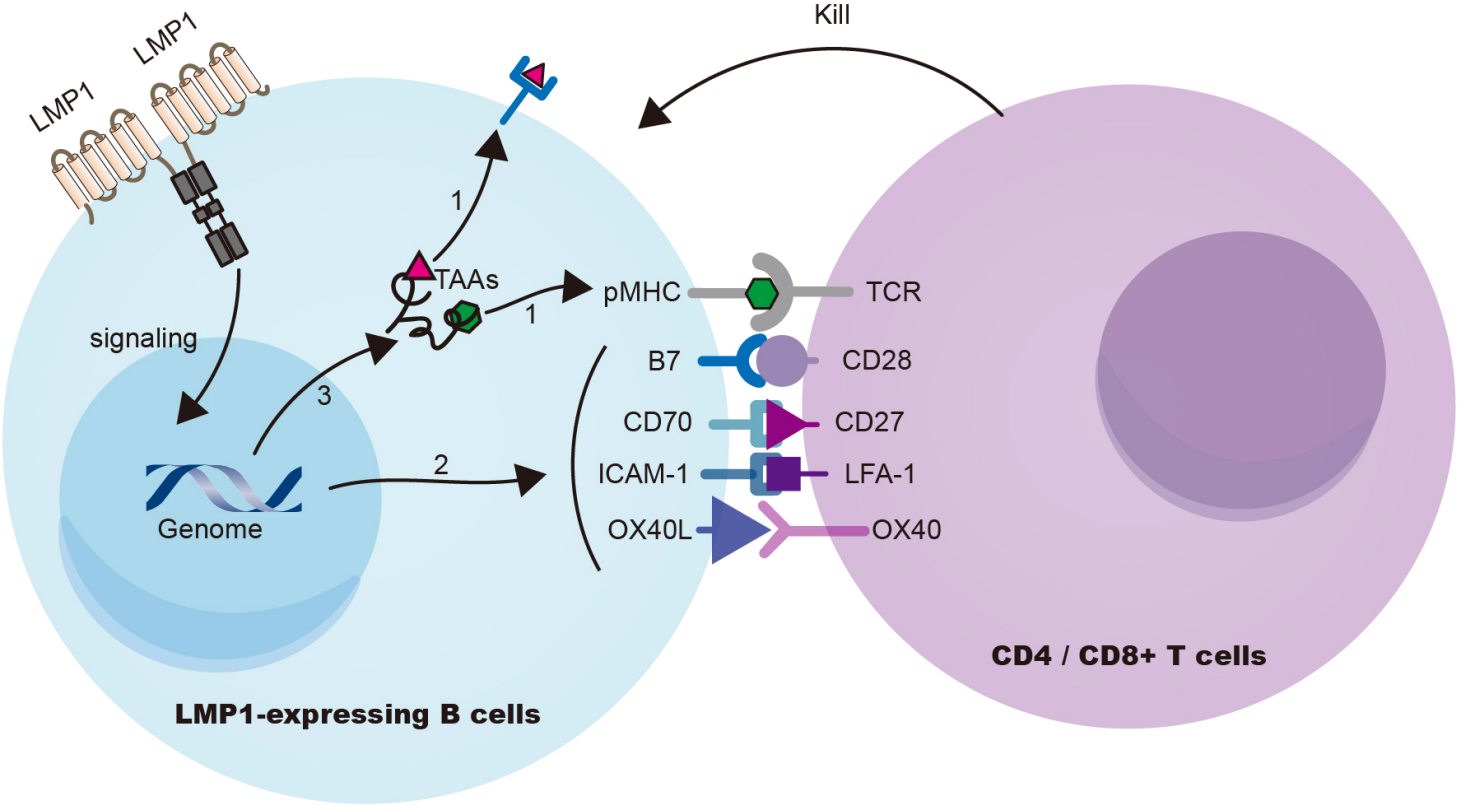
LMP1 signaling in B cells triggers cytotoxic T-cell resposes against TAAs. (Choi et al., 2021) LMP1 signaling induces substantial cellular gene expression, leading to (i) upregulation of antigen processing and presentation machinery, (ii) enhanced expression of co-stimulatory ligands (CD70, OX40L, etc.), and (iii) overexpression of cellular antigens known to function as TAAs. Collectively, these mechanisms contribute to the effective eradication of LMP1 (EBV)-transformed B cells. TAA, tumor associated antigens.

Choi et al. (10) demonstrated in a mouse model that the expression of the EBV signaling protein LMP1 in B cells induces T-cell responses against multiple TAAs. LMP1 signaling enhances the presentation of TAAs on B cells and upregulates the expression of costimulatory ligands CD70 and OX40L, leading to the activation of potent cytotoxic CD4+ and CD8+ T-cell responses against LMP1 (EBV)-transformed B cells (**Figure 2**). Furthermore, through the ectopic expression of LMP1 on patient-derived tumor B cells to prime T cells, autologous cytotoxic CD4+ T cells can be expanded to target a wide range of endogenous tumor antigens, including TAAs and neoantigens. This innovative approach holds great promise for treating B-cell malignancies and augmenting immune-mediated protection against EBV-unrelated cancers by targeting shared TAAs (96).

Several independent studies have also reported a nonviral, cellular antigen-specific component in the human CD4+ T cell response upon EBV-transformed LCL stimulation *in vitro* (39, 97). However, these cellular antigens have not been identified and their classification as TAAs remains to be established. Furthermore, clinical studies have observed the detection of T cells specific for nonviral TAAs in the peripheral blood following cytotoxic T lymphocyte (CTL) infusion, which is associated with clinical responses (85). Nevertheless, whether these T cells arise through epitope spreading or are derived from the therapeutic T cells through LCL stimulation is unclear. Therefore, further investigations are needed to identify TAAs expressed by EBV-infected or transformed B cells and to determine their recognition by T cells in individuals with EBV infection (96).

In addition to B cells, whether LMP1 or other EBV antigens can induce the upregulation of TAAs in epithelial cells has yet to be examined. Furthermore, the exact roles of MHC II molecules in cancer remain subject to debate and investigation. Accumulating evidence indicates that tumor-specific MHC II expression is linked to positive outcomes in many cancer types (98) (e.g., breast cancer (99), colon cancer (100), melanoma (101)). However, an opposing functional aspect of MHC II has also emerged. In HLA-DR+ melanoma, MHC II lessens CD8+ T cell activity by inducing LAG3+ and FCRL6+ TILs (102) or recruiting CD4+ T cells to the tumor (103). In the TC-1 mouse model of HPV-related carcinoma, the absence of MHC II molecules promotes CD8+ T cell infiltration and activation, curbing tumor growth (104). Moreover, a recent study examining NPC using single-cell transcriptomics has revealed a dual epithelial-immune feature of tumor cells, characterized by the expression of immune-related genes, including MHC II-coding genes (78), which relates to poor prognosis. This distinct trait also links to CD8+ T cell exhaustion and a suppressed tumor environment (78).

## 5 EBV-specific T cells in TME: bystanders or not?

Humans can experience common viral infections like CMV, EBV, and influenza. Once recovered, antiviral memory T cells are retained throughout the body to sense reinfection or recrudescence (105, 106) and are endowed with the capacity for rapid response, sustained vigilance, and cytotoxic prowess (107). Although such virus-specific T cells are abundant within tumors, they may not target tumor cells and are therefore regarded as “bystander-T cells” (108). However, emerging evidence suggests that these virus-specific T cells can still be harnessed for cancer immunotherapy (107, 109–111).

One strategy involves antibody-mediated delivery of viral epitopes to tumors (110, 111), achieved by conjugating virus-derived epitopes with tumor-targeting antibodies. These antibodies bind to specific tumor cell antigens and release immunogenic virus epitopes when cleaved by tumor-specific proteases. The released peptide then binds to free HLA class I molecules at the tumor cell’s surface and can be targeted for destruction by circulating virus-specific CTLs (110, 111).

Another strategy employs viral peptides to mimic a viral reinfection event in memory T cells. Memory T cells can execute a ‘sensing and alarm’ function upon antigen re-exposure (112), and this form of immunotherapy is termed peptide alarm therapy (PAT) (109). Reactivating virus-specific memory T cells through intratumoral delivery of adjuvant-free virus-derived peptide triggers local immune activation. This delivery translates to antineoplastic effects, which lead to a significant tumor reduction of tumor growth in mouse models of melanoma (107) and improved survival in a murine glioblastoma model (109). This approach can reactivate and attract T-cell infiltration into the tumor and transform the immunosuppressive tumor microenvironment into immune-active sites.

## 6 EBV-specific T cell-based therapies

### 6.1 EBVSTs

EBV-specific T cells (EBVSTs) derived from allogeneic or autologous donors can recognize and eliminate cancer cells expressing EBV antigens, highlighting their potential in adoptive cell therapy (**Table 1**).

**Table 1:** EBV-associated malignancies and their forms of viral latency.

tumor	subtype	% EBV positive	latency
PTLD	post HSCT	>95%	III
PTLD	post SOT	95% in first year	III
		50-60% after 1 year	I/II
Hodgkin’s lymphoma	classical	30-40%	II
	AIDS-related	100%	
Diffuse large B cell lymphoma	late post-transplant DLBCL	>50%	I/II
	Elderly DLBCL	>50%	
	AIDS-related	~50%	
Burkitt’s lymphoma	Endemic BL	100%	I
	AIDS-related BL	30-40%	
T/NK cell lymphoma	Extranodal	>95%	I/II
Nasopharygeal carcinoma	Undifferentiated	100%	I/II
Gastric carcinoma		9%	I/II

PTLD, post-transplant lymphoproliferative disease; HSCT, hematopoietic stem cell transplant; SOT, solid organ transplant; AIDS, acquired immunodeficiency syndrome; DLBCL, diffuse large B cell lymphoma; EBV, Epstein-Barr virus; NK, natural killer.

Clinical trials in the early stages have demonstrated the effectiveness of adoptive T-cell therapy in treating PTLD, which leverages the restoration of cellular immunity to control EBV-associated PTLD. Initial trials using unmanipulated donor-derived lymphocytes in HSCT patients yielded favorable outcomes, with complete regression observed in all 5 patients (113). However, the alloreactive nature of these T cells also led to the development of graft-versus-host disease (GvHD). Subsequent trials focused on generating allogeneic EBVSTs through *in vitro* stimulation using EBV-transformed LCLs, recombinant viral vectors, or synthetic peptides (86, 114–118). These trials demonstrated efficacy in preventing and treating PTLD in HSCT recipients, with minimal alloreactivity and reduced production pipeline. Similar strategies have been employed in the context of SOT to address PTLD (119–121); however, the response rate and persistence of EBVSTs in SOT patients have been limited, likely attributed to high levels of immunosuppression (9). To overcome this challenge, preclinical studies have attempted genetic modifications of EBVSTs to confer resistance against immunosuppressive agents (122–124).

The success of EBVSTs in PTLD has fostered an interest in treating other EBV-associated malignancies, such as NPC and HL. EBVSTs targeting type II latency antigens (EBNA1, LMP1, and LMP2) have shown promising results in clinical trials (85, 87, 88, 117, 125), with increased response rates and overall survival observed in patients with NPC and HL compared to those who did not receive adoptive cell transfer. However, it should be noted that the best response rate is still observed in PTLD-post HSCT (**Table 2**). In addition, emerging evidence indicates that immediate early and other lytic transcripts, including BARF1, could broaden specificity and enhance cytotoxicity for EBV-associated diseases. BARF1-specific T cells have demonstrated the ability to efficiently eliminate NPC cell lines *in vitro* (127).

**Table 2:** Summary of EBV-specific T cell-based therapies.

method	disease, clinical trial/animal model	phase	published year (reference)	results
Adoptive cell therapy				
Donor-derived unmanipulated lymphocytes	PTLD	I	1994 (113)	CR in 5/5 patients, but GVHD developed
gene-modified donor-derived EBVSTs	PTLD, HSCT	I	1995 (114)	CR of immunoblastic lymphoma in 1/1 patients; EBV reactivation controlled in 3/3 patients without lymphoma
Donor-derived EBVSTs	PTLD, HSCT	I	1998 (115)	CR of immunoblastic lymphoma in 2/2 patients; EBV reactivation controlled in 6/6 patients without lymphoma
Multivirus-specific CTLs activated by LCLs genetically modified with an adenoviral vector	PTLD, HSCT	I	2006 (116)	CR in 11/11 individuals with evidence of active CMV, EBV or adenoviral infection, without GVHD
EBV, ADV-specific CTLs activated by monocytes and LCLs transduced with adenoviral vector	PTLD, HSCT	I	2009 (118)	none of 13 receiving EBVSTs as prophylaxis developed PTLD
Peptide-induced multivirus-CTL	PTLD, HSCT	I	2014 (86)	11 recipients: 94% response rate of 8 patients receiving EBVSTs as treatment; 3 patients receiving EBVSTs as prophylaxis did not develop PTLD.
third party-EBVSTs	PTLD	II	2007 (38)	CR or PR in 17/33 patients
third party-EBVSTs	PTLD	I	2019 (126)	CR or PR in 35/59 patients
Autologous EBVSTs	PTLD, SOT	I	1999 (119)	Significant regression of the PTLD in 1/1 patient
Autologous EBVSTs	PTLD, SOT	I	2006 (120)	CR of liver PTLD in 1/1 patient; prevention of PTLD in 12/12 patients
Autologous EBVSTs	SOT	I	2002 (121)	Decrease EBV load, prevention of PTLD in 7/7 patients
Autologous EBVSTs	NPC	I	2005 (125)	CR in 4/10 patients and PR in 2/10 patients
Autologous EBVSTs combined with chemotherapy	NPC	II	2014 (87)	CR in 3/35 patients and PR in 22/35 patients
LMP1/LMP2-specific Autologous EBVSTs	Lymphoma	II	2014 (85)	CR in 11/21 patients and PR 2/21 patients
LMP1 and LMP2a-specific EBVSTs	Extranodal NK/T Cell Lymphoma	I	2015 (88)	CR in 10/10 patients
LMP- EBVST	HSCT	I	2018 (117)	PR in 2/7 post treatment therapy 15/19 remain in remission post prophylaxis therapy
BARF1-EBVST	NPC cell lines		2016 (127)	CTLs generated with doxorubicin-treated LCLs kill T2-A2 cells with exogenous or endogenous BARF1-peptides
EBV Specific TCR engineered T cell therapy				
Autologous CD8 and CD4 Lymphocytes expressing LMP2-specific TCR	NSG mouse, NPC model		2015 (128)	Lysed LMP2+ NPC cells and inhibited tumor growth in a mouse model
LMP1-specific TCR-T	NSG mouse, tumor model		2018 (129)	Doubled the survival time of mice bearing tumor
EBV-Specific γδ T cells				
Anti-γδ TCR antibody-expanded γδ T cells	nude mice, lymphoma model		2012 (130)	adoptive transfer of the expanded γδ T cells to Daudi lymphoma-bearing nude mice significantly prolonged the survival time of the mice and improved their living status
pamidronate-expanded Vγ9Vδ2-T cells	EBV-LPD Model in Humanized and Rag2–/– γc–/– Mice		2014 (131)	prevented and inhibited EBV-LPD in mouse models
adoptive γδ T cells combined with EBV-targeting probe (L2)P4	NPC-bearing NSG mice model		2023 (132)	exerted killing against certain NPC cells, enhanced tumor regression *in vivo* by adoptive transfer of γδ T cells
Exosomes derived from Vδ2-T cells	EBV-associated tumors in Rag2−/−δc−/− mice and humanized mice		2020 (133)	Vδ2-T-Exos induce CD4 and CD8 T cell–mediated antitumor immunity and control EBV-associated tumors in Rag2−/−δc−/− mice and humanized mice models.
γδ-T-Exos combined with radiotherapy	NPC tumors in Rag2−/−δc−/− mice		2022 (134)	γδ-T-Exos synergize with radiotherapy to control NPC tumors *in vivo*, and preserve antitumor activities in immunosuppressive NPC microenvironment

this list contains examples.

CR, complete response; PR, partial response.

To improve accessibility and expedite treatment, the establishment of third-party EBVST banks is actively being explored for PTLD (38, 126). The use of banked cells from third-party donors has broadened the availability of EBVSTs, and the observed response rates indicate the potential effectiveness of this approach in a wider range of patients. Alternatively, a combination of therapies with other immunomodulatory agents, such as checkpoint inhibitors (135) or vaccines (136) may be necessary to ensure clinical impact.

### 6.2 EBV specific T cell receptor engineered T cell therapy

TCR (T-cell receptor) engineered T-cell therapy has emerged as a promising strategy for immune-based treatment (**Table 1**). TCRs specific to EBNA3A, EBNA3B, LMP1, LMP2, BRLF1, and BMLF1 have been generated from CD8+ T cell clones (129, 137, 138). However, recognition of autologous EBV-transformed LCLs by T-cell lines transduced with these TCRs was weak, partly attributed to the limited expression of latent EBV antigens in LCLs. Nevertheless, the adoptive transfer of TCR transgenic T cells significantly attenuated tumor growth induced by the CNE NPC line in nude mice, demonstrating their efficacy *in vivo* (139). The interactions between transgenic TCR α and β chains with the endogenous TCR is another possible factor contributing to the constrained killing efficiency (140). To overcome this, chimeric TCRs have been devised. These chimeric TCRs entail the fusion of constant regions derived from mouse TCR with variable domains derived from EBV-specific T cell clones (141). The stability of these modified receptors was enhanced by introducing an additional disulfide bond between the TCR α and β chain constant domains (128, 142). Transgenic T cells expressing these chimeric TCRs exhibited improved cytotoxicity against co-incubated EBV-positive NPC cells, effectively suppressing tumor growth in immune-compromised mice (128). Similarly, promising outcomes were observed with an LMP1-specific TCR, as T cells transduced with LMP1-specific TCR rendered a twofold increase in the survival of immune-compromised mice challenged with LMP1-expressing tumor cells (129).

Consequently, despite the limited cytotoxicity towards autologous tumor cells, transgenic T-cell therapy remains a promising strategy in combating EBV-associated malignancies.

## 7 Beyond αβ: accumulating evidence of a role for γδ T-cells

The preceding review primarily focuses on αβ T cells, but it is important to note the unique features of γδ T cells that make them appealing in various cancer settings. These features include tissue tropisms, MHC-independent antigen presentation, antitumor activity regardless of neoantigen burden (143), and a combination of T and natural killer cell properties (144–146). In humans, γδ T cells can be categorized into Vδ1+ and Vδ2+ cells, with distinct distributions in mucosal tissues and blood/lymphoid organs, respectively. They play a crucial role in antiviral immune responses in cytomegalovirus (147–150). Emerging evidence suggests that γδ T cells also play a role in primary EBV infection and EBV-associated cancers.

During primary EBV infection, there is an observed increase in the frequency of γδ T cells in the blood of patients with infectious mononucleosis (IM) (151–153). Pediatric patients have a bimodal innate response to primary EBV infection (154), influenced by a dimorphism in TCRγ-chain repertoires (155). Altered γδ T cells have also been observed in patients with EBV-associated malignancies, such as NPC, where the impaired functional capacity of γδ T cells is observed despite an unchanged frequency (156, 157). In a case involving a cord blood transplant recipient with elevated EBV viremia, the absence of detectable αβ T cells was compensated by expansions of cytotoxic Vδ1+ γδ T cells, resulting in no signs of lymphoproliferative disorder (158). Moreover, early recovery of Vδ2+ T cells has been identified as an independent protective factor against EBV reactivation in recipients of allo-HSCT (159). Interventions that induce early reconstitution of autologous γδ T cells could hold therapeutic benefits. αβ TCR graft depletion (160, 161) has demonstrated efficacy in reducing GVHD by facilitating rapid immune reconstitution of NK cells and γδ T cells (162–164). Additionally, reducing immunosuppressants has led to enhanced recovery of Vδ2+ T cells and decreased risk of EBV-associated lymphoproliferative disorders in HSCT recipients (159). Notably, long-term persistence of donor-derived Vδ1+ T cell clones has been detected in recipients’ blood even a decade post-HSCT, with these cells exhibiting expandability *in vitro* and cytotoxicity against autologous EBV-LCL (165).

While extensive research and clinical trials have explored the therapeutic potential of γδ T cells in managing solid tumors and hematopoietic malignancies (166–168), only a limited number of studies have investigated their efficacy in EBV-associated cancers using murine models (**Table 1**). Adoptive transfer of anti-γδ TCR antibody-expanded γδ T cells to Daudi lymphoma-bearing nude mice significantly prolonged their survival time (130). In addition, the adoptive transfer of pamidronate-expanded Vγ9Vδ2-T cells prevented and inhibited EBV-LPD in mouse models (131). Moreover, co-administration of Vδ2+ T cells and the EBNA1-targeting peptide L2P4 enhanced γδ T cell cytotoxicity against NPC in immunodeficient mouse models (132). Additionally, exosomes derived from Vδ2+ T cells exhibited the ability to eliminate EBV-associated tumor cells (133), and when combined with radiotherapy, γδ-T-Exos demonstrated efficacy in effectively treating NPC by eradicating radioresistant cells (134).

Thus, γδ T cells represent an essential component of cellular immunity in regulating primary EBV infection and hold promise in combating EBV-associated malignancies.

## 8 Conclusions

Cellular immunity is pivotal in maintaining the delicate equilibrium between the host and EBV. Despite EBV’s high prevalence, affecting a significant portion of the global population, most individuals remain asymptomatic throughout their lives, highlighting the critical role of effective immune control. However, EBV-associated malignancies primarily occur in individuals with apparently intact immune function. This raises intriguing questions about the mechanisms and stages at which these tumors manage to evade the surveillance of virus-specific T cells.

EBV-associated malignancies express distinct EBV latent antigens, triggering diverse T-cell responses while also employing a range of immune evasion mechanisms, rendering a complex interplay with cellular immunity. Encouragingly, promising clinical responses have been observed from adoptive cell transfer of EBV-specific T cells targeting latent antigens. Recent investigations into early lytic EBV antigens in tumorigenesis provide additional potential targets for therapeutic interventions. Additionally, TCR transgenic therapy offers the possibility of redirecting T cells to recognize EBV antigens and the involvement of γδ T cells also merits consideration in EBV-associated diseases.

In cancers not associated with EBV, there usually exists an abundance of EBV-specific memory T cells, which can be leveraged to either activate the immunosuppressive tumor microenvironment or re-directed to target tumor cells. In addition, EBV can activate TAA-specific T-cell responses. These further broaden our understanding of this oncogenic virus and its implications for the fields of cancer biology and therapy. In this regard, a pivotal research goal is to attain a comprehensive grasp of the intricate interplay between cellular immunity and the virus. By harnessing the inherent capabilities of T-cell immunity, we can advance toward more precise and effective interventions in the treatment of EBV-associated and other cancers.

## Author contributions

QZ prepared the manuscript draft, MX provided valuable comments to the text and figures, read and approved the final manuscript. All authors contributed to the article and approved the submitted version.

## References

[1] Arvin A Campadelli-Fiume G Mocarski E Moore PS Roizman B Whitley R Yamanishi K eds. Human herpesviruses: biology, therapy, and immunoprophylaxis. Cambridge: Cambridge University Press (2007). 21348071

[2] AbuSalah MAH Gan SH Al-Hatamleh MAI Irekeola AA Shueb RH Yean Yean C. Recent advances in diagnostic approaches for epstein-barr virus. Pathog Basel Switz (2020) 9:226. doi: 10.3390/pathogens9030226 PMC7157745 32197545

[3] Wong Y Meehan MT Burrows SR Doolan DL Miles JJ. Estimating the global burden of Epstein–Barr virus-related cancers. J Cancer Res Clin Oncol (2022) 148:31–46. doi: 10.1007/s00432-021-03824-y 34705104 PMC8752571

[4] Dharnidharka VR. Comprehensive review of post-organ transplant hematologic cancers. Am J Transplant Off J Am Soc Transplant Am Soc Transpl Surg (2018) 18:537–49. doi: 10.1111/ajt.14603 29178667

[5] Al-Mansour Z Nelson BP Evens AM. Post-transplant lymphoproliferative disease (PTLD): risk factors, diagnosis, and current treatment strategies. Curr Hematol Malig Rep (2013) 8:173–83. doi: 10.1007/s11899-013-0162-5 PMC4831913 23737188

[6] AlDabbagh MA Gitman MR Kumar D Humar A Rotstein C Husain S. The role of antiviral prophylaxis for the prevention of epstein-barr virus-associated posttransplant lymphoproliferative disease in solid organ transplant recipients: A systematic review. Am J Transplant Off J Am Soc Transplant Am Soc Transpl Surg (2017) 17:770–81. doi: 10.1111/ajt.14020 27545492

[7] Tangye SG Palendira U Edwards ESJ. Human immunity against EBV-lessons from the clinic. J Exp Med (2017) 214:269–83. doi: 10.1084/jem.20161846 PMC5294862 28108590

[8] Hislop AD Taylor GS Sauce D Rickinson AB. Cellular responses to viral infection in humans: lessons from Epstein-Barr virus. Annu Rev Immunol (2007) 25:587–617. doi: 10.1146/annurev.immunol.25.022106.141553 17378764

[9] Heslop HE Sharma S Rooney CM. Adoptive T-cell therapy for epstein-barr virus-related lymphomas. J Clin Oncol Off J Am Soc Clin Oncol (2021) 39:514–24. doi: 10.1200/JCO.20.01709 PMC8462582 33434061

[10] Choi I-K Wang Z Ke Q Hong M Paul DWJ Fernandes SM. Mechanism of EBV inducing anti-tumour immunity and its therapeutic use. Nature (2021) 590:157–62. doi: 10.1038/s41586-020-03075-w PMC7864874 33361812

[11] Cohen JI. Epstein-Barr virus infection. N Engl J Med (2000) 343:481–92. doi: 10.1056/NEJM200008173430707 10944566

[12] Thorley-Lawson DA Hawkins JB Tracy SI Shapiro M. The pathogenesis of Epstein-Barr virus persistent infection. Curr Opin Virol (2013) 3:227–32. doi: 10.1016/j.coviro.2013.04.005 PMC3789532 23683686

[13] Taylor GS Long HM Brooks JM Rickinson AB Hislop AD. The immunology of Epstein-Barr virus-induced disease. Annu Rev Immunol (2015) 33:787–821. doi: 10.1146/annurev-immunol-032414-112326 25706097

[14] Odumade OA Hogquist KA Balfour HH. Progress and problems in understanding and managing primary Epstein-Barr virus infections. Clin Microbiol Rev (2011) 24:193–209. doi: 10.1128/CMR.00044-10 21233512 PMC3021204

[15] Murata T. Encyclopedia of EBV-encoded lytic genes: an update. In: Kawaguchi Y Mori Y Kimura H, editors. Human herpesviruses. Advances in experimental medicine and biology. Singapore: Springer (2018). p. 395–412. doi: 10.1007/978-981-10-7230-7_18 29896677

[16] Kelly GL Long HM Stylianou J Thomas WA Leese A Bell AI. An Epstein-Barr virus anti-apoptotic protein constitutively expressed in transformed cells and implicated in burkitt lymphomagenesis: the Wp/BHRF1 link. PloS Pathog (2009) 5:e1000341. doi: 10.1371/journal.ppat.1000341 19283066 PMC2652661

[17] Sausen DG Bhutta MS Gallo ES Dahari H Borenstein R. Stress-induced epstein-barr virus reactivation. Biomolecules (2021) 11:1380. doi: 10.3390/biom11091380 34572593 PMC8470332

[18] Ternák G. Epstein-Barr virus reactivation. Lancet Infect Dis (2003) 3:271. doi: 10.1016/s1473-3099(03)00603-0 12726972

[19] Simonnet A Engelmann I Moreau A-S Garcia B Six S El Kalioubie A. High incidence of Epstein-Barr virus, cytomegalovirus, and human-herpes virus-6 reactivations in critically ill patients with COVID-19. Infect Dis Now (2021) 51:296–9. doi: 10.1016/j.idnow.2021.01.005 PMC7816954 33495765

[20] Makielski KR Lee D Lorenz LD Nawandar DM Chiu Y-F Kenney SC. Human papillomavirus promotes Epstein-Barr virus maintenance and lytic reactivation in immortalized oral keratinocytes. Virology (2016) 495:52–62. doi: 10.1016/j.virol.2016.05.005 27179345 PMC4912861

[21] Cao P Zhang M Wang L Sai B Tang J Luo Z. miR-18a reactivates the Epstein-Barr virus through defective DNA damage response and promotes genomic instability in EBV-associated lymphomas. BMC Cancer (2018) 18:1293. doi: 10.1186/s12885-018-5205-9 30594162 PMC6311029

[22] Schmeer KK Ford JL Browning CR. Early childhood family instability and immune system dysregulation in adolescence. Psychoneuroendocrinology (2019) 102:189–95. doi: 10.1016/j.psyneuen.2018.12.014 PMC6689237 30579236

[23] Murray RJ Kurilla MG Brooks JM Thomas WA Rowe M Kieff E. Identification of target antigens for the human cytotoxic T cell response to Epstein-Barr virus (EBV): implications for the immune control of EBV-positive Malignancies. J Exp Med (1992) 176:157–68. doi: 10.1084/jem.176.1.157 PMC2119296 1319456

[24] Steven NM Leese AM Annels NE Lee SP Rickinson AB. Epitope focusing in the primary cytotoxic T cell response to Epstein-Barr virus and its relationship to T cell memory. J Exp Med (1996) 184:1801–13. doi: 10.1084/jem.184.5.1801 PMC2192864 8920868

[25] Morales-Sánchez A Fuentes-Panana EM. The immunomodulatory capacity of an epstein-barr virus abortive lytic cycle: potential contribution to viral tumorigenesis. Cancers (2018) 10:98. doi: 10.3390/cancers10040098 29601503 PMC5923353

[26] Kalla M Schmeinck A Bergbauer M Pich D Hammerschmidt W. AP-1 homolog BZLF1 of Epstein-Barr virus has two essential functions dependent on the epigenetic state of the viral genome. Proc Natl Acad Sci U.S.A. (2010) 107:850–5. doi: 10.1073/pnas.0911948107 PMC2818922 20080764

[27] Shannon-Lowe C Adland E Bell AI Delecluse H-J Rickinson AB Rowe M. Features distinguishing Epstein-Barr virus infections of epithelial cells and B cells: viral genome expression, genome maintenance, and genome amplification. J Virol (2009) 83:7749–60. doi: 10.1128/JVI.00108-09 PMC2708605 19439479

[28] Inagaki T Sato Y Ito J Takaki M Okuno Y Yaguchi M. Direct evidence of abortive lytic infection-mediated establishment of epstein-barr virus latency during B-cell infection. Front Microbiol (2020) 11:575255. doi: 10.3389/fmicb.2020.575255 33613459 PMC7888302

[29] Wen W Iwakiri D Yamamoto K Maruo S Kanda T Takada K. Epstein-Barr virus BZLF1 gene, a switch from latency to lytic infection, is expressed as an immediate-early gene after primary infection of B lymphocytes. J Virol (2007) 81:1037–42. doi: 10.1128/JVI.01416-06 PMC1797481 17079287

[30] Tsang CM Zhang G Seto E Takada K Deng W Yip YL. Epstein-Barr virus infection in immortalized nasopharyngeal epithelial cells: regulation of infection and phenotypic characterization. Int J Cancer (2010) 127:1570–83. doi: 10.1002/ijc.25173 20091869

[31] Ramayanti O Juwana H Verkuijlen SAMW Adham M Pegtel MD Greijer AE. Epstein-Barr virus mRNA profiles and viral DNA methylation status in nasopharyngeal brushings from nasopharyngeal carcinoma patients reflect tumor origin. Int J Cancer (2017) 140:149–62. doi: 10.1002/ijc.30418 PMC5129462 27600027

[32] Nawandar DM Wang A Makielski K Lee D Ma S Barlow E. Differentiation-dependent KLF4 expression promotes lytic epstein-barr virus infection in epithelial cells. PloS Pathog (2015) 11:e1005195. doi: 10.1371/journal.ppat.1005195 26431332 PMC4592227

[33] Vrzalikova K Vockerodt M Leonard S Bell A Wei W Schrader A. Down-regulation of BLIMP1α by the EBV oncogene, LMP-1, disrupts the plasma cell differentiation program and prevents viral replication in B cells: implications for the pathogenesis of EBV-associated B-cell lymphomas. Blood (2011) 117:5907–17. doi: 10.1182/blood-2010-09-307710 PMC3293751 21411757

[34] Hu L Lin Z Wu Y Dong J Zhao B Cheng Y. Comprehensive profiling of EBV gene expression in nasopharyngeal carcinoma through paired-end transcriptome sequencing. Front Med (2016) 10:61–75. doi: 10.1007/s11684-016-0436-0 26969667

[35] Ma S-D Hegde S Young KH Sullivan R Rajesh D Zhou Y. A new model of Epstein-Barr virus infection reveals an important role for early lytic viral protein expression in the development of lymphomas. J Virol (2011) 85:165–77. doi: 10.1128/JVI.01512-10 PMC3014199 20980506

[36] Hong GK Gulley ML Feng W-H Delecluse H-J Holley-Guthrie E Kenney SC. Epstein-Barr virus lytic infection contributes to lymphoproliferative disease in a SCID mouse model. J Virol (2005) 79:13993–4003. doi: 10.1128/JVI.79.22.13993-14003.2005 PMC1280209 16254335

[37] Nikiforow S Bottomly K Miller G. CD4+ T-cell effectors inhibit epstein-barr virus-induced B-cell proliferation. J Virol (2001) 75:3740–52. doi: 10.1128/JVI.75.8.3740-3752.2001 PMC114865 11264363

[38] Haque T Wilkie GM Jones MM Higgins CD Urquhart G Wingate P. Allogeneic cytotoxic T-cell therapy for EBV-positive posttransplantation lymphoproliferative disease: results of a phase 2 multicenter clinical trial. Blood (2007) 110:1123–31. doi: 10.1182/blood-2006-12-063008 17468341

[39] Long HM Zuo J Leese AM Gudgeon NH Jia H Taylor GS. CD4+ T-cell clones recognizing human lymphoma-associated antigens: generation by in *vitro* stimulation with autologous Epstein-Barr virus-transformed B cells. Blood (2009) 114:807–15. doi: 10.1182/blood-2008-12-194043 19443664

[40] Gudgeon NH Taylor GS Long HM Haigh TA Rickinson AB. Regression of Epstein-Barr virus-induced B-cell transformation in *vitro* involves virus-specific CD8+ T cells as the principal effectors and a novel CD4+ T-cell reactivity. J Virol (2005) 79:5477–88. doi: 10.1128/JVI.79.9.5477-5488.2005 PMC1082738 15827162

[41] Levitskaya J Coram M Levitsky V Imreh S Steigerwald-Mullen PM Klein G. Inhibition of antigen processing by the internal repeat region of the Epstein-Barr virus nuclear antigen-1. Nature (1995) 375:685–8. doi: 10.1038/375685a0 7540727

[42] Voo KS Fu T Wang HY Tellam J Heslop HE Brenner MK. Evidence for the presentation of major histocompatibility complex class I-restricted Epstein-Barr virus nuclear antigen 1 peptides to CD8+ T lymphocytes. J Exp Med (2004) 199:459–70. doi: 10.1084/jem.20031219 PMC2211826 14769850

[43] Tellam J Connolly G Green KJ Miles JJ Moss DJ Burrows SR. Endogenous presentation of CD8+ T cell epitopes from Epstein-Barr virus-encoded nuclear antigen 1. J Exp Med (2004) 199:1421–31. doi: 10.1084/jem.20040191 PMC2211806 15148340

[44] Lee SP Brooks JM Al-Jarrah H Thomas WA Haigh TA Taylor GS. CD8 T cell recognition of endogenously expressed epstein-barr virus nuclear antigen 1. J Exp Med (2004) 199:1409–20. doi: 10.1084/jem.20040121 PMC2211813 15148339

[45] Lee SP Constandinou CM Thomas WA Croom-Carter D Blake NW Murray PG. Antigen presenting phenotype of Hodgkin Reed-Sternberg cells: analysis of the HLA class I processing pathway and the effects of interleukin-10 on epstein-barr virus-specific cytotoxic T-cell recognition. Blood (1998) 92:1020–30. doi: 10.1182/blood.V92.3.1020.415a20_1020_1030 9680372

[46] Murray PG Constandinou CM Crocker J Young LS Ambinder RF. Analysis of major histocompatibility complex class I, TAP expression, and LMP2 epitope sequence in Epstein-Barr virus-positive Hodgkin’s disease. Blood (1998) 92:2477–83. doi: 10.1182/blood.V92.7.2477 9746788

[47] Oudejans JJ Jiwa NM Kummer JA Horstman A Vos W Baak JP. Analysis of major histocompatibility complex class I expression on Reed-Sternberg cells in relation to the cytotoxic T-cell response in Epstein-Barr virus-positive and -negative Hodgkin’s disease. Blood (1996) 87:3844–51. doi: 10.1182/blood.V87.9.3844.bloodjournal8793844 8611711

[48] Fox CP Haigh TA Taylor GS Long HM Lee SP Shannon-Lowe C. A novel latent membrane 2 transcript expressed in Epstein-Barr virus-positive NK- and T-cell lymphoproliferative disease encodes a target for cellular immunotherapy. Blood (2010) 116:3695–704. doi: 10.1182/blood-2010-06-292268 PMC2981530 20671118

[49] Lee SP Chan AT Cheung ST Thomas WA CroomCarter D Dawson CW. CTL control of EBV in nasopharyngeal carcinoma (NPC): EBV-specific CTL responses in the blood and tumors of NPC patients and the antigen-processing function of the tumor cells. J Immunol Baltim Md 1950 (2000) 165:573–82. doi: 10.4049/jimmunol.165.1.573 10861098

[50] Masucci MG Stam NJ Torsteinsdottir S Neefjes JJ Klein G Ploegh HL. Allele-specific down-regulation of MHC class I antigens in Burkitt lymphoma lines. Cell Immunol (1989) 120:396–400. doi: 10.1016/0008-8749(89)90207-4 2541930

[51] Khanna R Burrows SR Thomson SA Moss DJ Cresswell P Poulsen LM. Class I processing-defective Burkitt’s lymphoma cells are recognized efficiently by CD4+ EBV-specific CTLs. J Immunol Baltim Md 1950 (1997) 158:3619–25. doi: 10.4049/jimmunol.158.8.3619 9103423

[52] Fu T Voo KS Wang R-F. Critical role of EBNA1-specific CD4+ T cells in the control of mouse Burkitt lymphoma *in vivo* . J Clin Invest (2004) 114:542–50. doi: 10.1172/JCI22053 PMC503775 15314691

[53] Paludan C Bickham K Nikiforow S Tsang ML Goodman K Hanekom WA. Epstein-Barr nuclear antigen 1-specific CD4(+) Th1 cells kill Burkitt’s lymphoma cells. J Immunol Baltim Md 1950 (2002) 169:1593–603. doi: 10.4049/jimmunol.169.3.1593 12133989

[54] Bei J-X Li Y Jia W-H Feng B-J Zhou G Chen L-Z. A genome-wide association study of nasopharyngeal carcinoma identifies three new susceptibility loci. Nat Genet (2010) 42:599–603. doi: 10.1038/ng.601 20512145

[55] Tse K-P Su W-H Chang K-P Tsang N-M Yu C-J Tang P. Genome-wide association study reveals multiple nasopharyngeal carcinoma-associated loci within the HLA region at chromosome 6p21.3. Am J Hum Genet (2009) 85:194–203. doi: 10.1016/j.ajhg.2009.07.007 19664746 PMC2725267

[56] Bei J-X Jia W-H Zeng Y-X. Familial and large-scale case-control studies identify genes associated with nasopharyngeal carcinoma. Semin Cancer Biol (2012) 22:96–106. doi: 10.1016/j.semcancer.2012.01.012 22313875

[57] Lu SJ Day NE Degos L Lepage V Wang PC Chan SH. Linkage of a nasopharyngeal carcinoma susceptibility locus to the HLA region. Nature (1990) 346:470–1. doi: 10.1038/346470a0 2377207

[58] Murray PG Young LS. An etiological role for the Epstein-Barr virus in the pathogenesis of classical Hodgkin lymphoma. Blood (2019) 134:591–6. doi: 10.1182/blood.2019000568 31186275

[59] Moss DJ Burrows SR Castelino DJ Kane RG Pope JH Rickinson AB. A comparison of Epstein-Barr virus-specific T-cell immunity in malaria-endemic and -nonendemic regions of Papua New Guinea. Int J Cancer (1983) 31:727–32. doi: 10.1002/ijc.2910310609 6305850

[60] Whittle HC Brown J Marsh K Greenwood BM Seidelin P Tighe H. T-cell control of Epstein-Barr virus-infected B cells is lost during P. falciparum malaria. Nature (1984) 312:449–50. doi: 10.1038/312449a0 6095104

[61] López C Burkhardt B Chan JKC Leoncini L Mbulaiteye SM Ogwang MD. Burkitt lymphoma. Nat Rev Dis Primer (2022) 8:78. doi: 10.1038/s41572-022-00404-3 36522349

[62] Chen Y-P Chan ATC Le Q-T Blanchard P Sun Y Ma J. Nasopharyngeal carcinoma. Lancet Lond Engl (2019) 394:64–80. doi: 10.1016/S0140-6736(19)30956-0 31178151

[63] Saito M Kono K. Landscape of EBV-positive gastric cancer. Gastric Cancer (2021) 24:983–9. doi: 10.1007/s10120-021-01215-3 34292431

[64] Chetaille B Bertucci F Finetti P Esterni B Stamatoullas A Picquenot JM. Molecular profiling of classical Hodgkin lymphoma tissues uncovers variations in the tumor microenvironment and correlations with EBV infection and outcome. Blood (2009) 113:2765–3775. doi: 10.1182/blood-2008-07-168096 19096012

[65] Barros MHM Vera-Lozada G Soares FA Niedobitek G Hassan R. Tumor microenvironment composition in pediatric classical Hodgkin lymphoma is modulated by age and Epstein-Barr virus infection. Int J Cancer (2012) 131:1142–52. doi: 10.1002/ijc.27314 22025264

[66] Morales O Mrizak D François V Mustapha R Miroux C Depil S. Epstein-Barr virus infection induces an increase of T regulatory type 1 cells in Hodgkin lymphoma patients. Br J Haematol (2014) 166:875–90. doi: 10.1111/bjh.12980 25041527

[67] Assis MCG Campos AHFM Oliveira JSR Soares FA Silva JMK Silva PB. Increased expression of CD4+CD25 +FOXP3+ regulatory T cells correlates with Epstein-Barr virus and has no impact on survival in patients with classical Hodgkin lymphoma in Brazil. Med Oncol Northwood Lond Engl (2012) 29:3614–9. doi: 10.1007/s12032-012-0299-4 22791223

[68] Baumforth KRN Birgersdotter A Reynolds GM Wei W Kapatai G Flavell JR. Expression of the Epstein-Barr virus-encoded Epstein-Barr virus nuclear antigen 1 in Hodgkin’s lymphoma cells mediates Up-regulation of CCL20 and the migration of regulatory T cells. Am J Pathol (2008) 173:195–204. doi: 10.2353/ajpath.2008.070845 18502823 PMC2438297

[69] Yamamoto R Nishikori M Kitawaki T Sakai T Hishizawa M Tashima M. PD-1-PD-1 ligand interaction contributes to immunosuppressive microenvironment of Hodgkin lymphoma. Blood (2008) 111:3220–4. doi: 10.1182/blood-2007-05-085159 18203952

[70] Juszczynski P Ouyang J Monti S Rodig SJ Takeyama K Abramson J. The AP1-dependent secretion of galectin-1 by Reed Sternberg cells fosters immune privilege in classical Hodgkin lymphoma. Proc Natl Acad Sci U.S.A. (2007) 104:13134–9. doi: 10.1073/pnas.0706017104 PMC1936978 17670934

[71] Green MR Rodig S Juszczynski P Ouyang J Sinha P O’Donnell E. Constitutive AP-1 activity and EBV infection induce PD-L1 in Hodgkin lymphomas and posttransplant lymphoproliferative disorders: implications for targeted therapy. Clin Cancer Res Off J Am Assoc Cancer Res (2012) 18:1611–8. doi: 10.1158/1078-0432.CCR-11-1942 PMC3321508 22271878

[72] Yip WK Abdullah MA Yusoff SM Seow HF. Increase in tumour-infiltrating lymphocytes with regulatory T cell immunophenotypes and reduced zeta-chain expression in nasopharyngeal carcinoma patients. Clin Exp Immunol (2009) 155:412–22. doi: 10.1111/j.1365-2249.2008.03793.x PMC2669517 19220831

[73] Li J Huang Z-F Xiong G Mo H-Y Qiu F Mai H-Q. Distribution, characterization, and induction of CD8+ regulatory T cells and IL-17-producing CD8+ T cells in nasopharyngeal carcinoma. J Transl Med (2011) 9:189. doi: 10.1186/1479-5876-9-189 22051182 PMC3223152

[74] Chen Y-P Lv J-W Mao Y-P Li X-M Li J-Y Wang Y-Q. Unraveling tumour microenvironment heterogeneity in nasopharyngeal carcinoma identifies biologically distinct immune subtypes predicting prognosis and immunotherapy responses. Mol Cancer (2021) 20:14. doi: 10.1186/s12943-020-01292-5 33430876 PMC7798236

[75] Gong L Kwong DL-W Dai W Wu P Li S Yan Q. Comprehensive single-cell sequencing reveals the stromal dynamics and tumor-specific characteristics in the microenvironment of nasopharyngeal carcinoma. Nat Commun (2021) 12:1540. doi: 10.1038/s41467-021-21795-z 33750785 PMC7943808

[76] Liu Y He S Wang X-L Peng W Chen Q-Y Chi D-M. Tumour heterogeneity and intercellular networks of nasopharyngeal carcinoma at single cell resolution. Nat Commun (2021) 12:741. doi: 10.1038/s41467-021-21043-4 33531485 PMC7854640

[77] Chen Y-P Yin J-H Li W-F Li H-J Chen D-P Zhang C-J. Single-cell transcriptomics reveals regulators underlying immune cell diversity and immune subtypes associated with prognosis in nasopharyngeal carcinoma. Cell Res (2020) 30:1024–42. doi: 10.1038/s41422-020-0374-x PMC7784929 32686767

[78] Jin S Li R Chen M-Y Yu C Tang L-Q Liu Y-M. Single-cell transcriptomic analysis defines the interplay between tumor cells, viral infection, and the microenvironment in nasopharyngeal carcinoma. Cell Res (2020) 30:950–65. doi: 10.1038/s41422-020-00402-8 PMC7784966 32901110

[79] Naseem M Barzi A Brezden-Masley C Puccini A Berger MD Tokunaga R. Outlooks on Epstein-Barr virus associated gastric cancer. Cancer Treat Rev (2018) 66:15–22. doi: 10.1016/j.ctrv.2018.03.006 29631196 PMC5964025

[80] van Beek J zur Hausen A Klein Kranenbarg E van de Velde CJH Middeldorp JM van den Brule AJC. EBV-positive gastric adenocarcinomas: a distinct clinicopathologic entity with a low frequency of lymph node involvement. J Clin Oncol Off J Am Soc Clin Oncol (2004) 22:664–70. doi: 10.1200/JCO.2004.08.061 14966089

[81] Kang BW Seo AN Yoon S Bae HI Jeon SW Kwon OK. Prognostic value of tumor-infiltrating lymphocytes in Epstein-Barr virus-associated gastric cancer. Ann Oncol Off J Eur Soc Med Oncol (2016) 27:494–501. doi: 10.1093/annonc/mdv610 26673353

[82] Kuzushima K Nakamura S Nakamura T Yamamura Y Yokoyama N Fujita M. Increased frequency of antigen-specific CD8+ cytotoxic T lymphocytes infiltrating an Epstein-Barr virus–associated gastric carcinoma. J Clin Invest (1999) 104:163–71. doi: 10.1172/JCI6062 PMC408473 10411545

[83] Strong MJ Xu G Coco J Baribault C Vinay DS Lacey MR. Differences in gastric carcinoma microenvironment stratify according to EBV infection intensity: implications for possible immune adjuvant therapy. PloS Pathog (2013) 9:e1003341. doi: 10.1371/journal.ppat.1003341 23671415 PMC3649992

[84] Cancer Genome Atlas Research Network. Comprehensive molecular characterization of gastric adenocarcinoma. Nature (2014) 513:202–9. doi: 10.1038/nature13480 PMC4170219 25079317

[85] Bollard CM Gottschalk S Torrano V Diouf O Ku S Hazrat Y. Sustained complete responses in patients with lymphoma receiving autologous cytotoxic T lymphocytes targeting Epstein-Barr virus latent membrane proteins. J Clin Oncol Off J Am Soc Clin Oncol (2014) 32:798–808. doi: 10.1200/JCO.2013.51.5304 PMC3940538 24344220

[86] Papadopoulou A Gerdemann U Katari UL Tzannou I Liu H Martinez C. Activity of broad-spectrum T cells as treatment for AdV, EBV, CMV, BKV, and HHV6 infections after HSCT. Sci Transl Med (2014) 6:242ra83. doi: 10.1126/scitranslmed.3008825 PMC4181611 24964991

[87] Chia W-K Teo M Wang W-W Lee B Ang S-F Tai W-M. Adoptive T-cell transfer and chemotherapy in the first-line treatment of metastatic and/or locally recurrent nasopharyngeal carcinoma. Mol Ther J Am Soc Gene Ther (2014) 22:132–9. doi: 10.1038/mt.2013.242 PMC3978790 24297049

[88] Cho S-G Kim N Sohn H-J Lee SK Oh ST Lee H-J. Long-term outcome of extranodal NK/T cell lymphoma patients treated with postremission therapy using EBV LMP1 and LMP2a-specific CTLs. Mol Ther J Am Soc Gene Ther (2015) 23:1401–9. doi: 10.1038/mt.2015.91 PMC4817864 26017177

[89] Li YY Chung GTY Lui VWY To K-F Ma BBY Chow C. Exome and genome sequencing of nasopharynx cancer identifies NF-κB pathway activating mutations. Nat Commun (2017) 8:14121. doi: 10.1038/ncomms14121 28098136 PMC5253631

[90] Liu Z Derkach A Yu KJ Yeager M Chang Y-S Chen C-J. Patterns of human leukocyte antigen class I and class II associations and cancer. Cancer Res (2021) 81:1148–52. doi: 10.1158/0008-5472.CAN-20-2292 PMC9986718 33272927

[91] Tang M Zeng Y Poisson A Marti D Guan L Zheng Y. Haplotype-dependent HLA susceptibility to nasopharyngeal carcinoma in a Southern Chinese population. Genes Immun (2010) 11:334–42. doi: 10.1038/gene.2009.109 PMC3737777 20072141

[92] Kirimunda S Verboom M Otim I Ssennono M Legason ID Nabalende H. Variation in the human leukocyte antigen (HLA) system and risk for endemic Burkitt Lymphoma in Northern Uganda. Br J Haematol (2020) 189:489–99. doi: 10.1111/bjh.16398 PMC7192769 32072624

[93] VietzenHFurlanoPLCornelissenJJBöhmigGAJakschPPuchhammer-StöcklE. HLA-E-restricted immune responses are crucial for the control of EBV infections and the prevention of PTLD. Blood (2023) 141:1560–73. doi: 10.1182/blood.2022017650 36477802

[94] Cai M-B Han H-Q Bei J-X Liu C-C Lei J-J Cui Q. Expression of human leukocyte antigen G is associated with prognosis in nasopharyngeal carcinoma. Int J Biol Sci (2012) 8:891–900. doi: 10.7150/ijbs.4383 22745579 PMC3385011

[95] Zhou X Cao S-M Cai Y-L Zhang X Zhang S Feng G-F. A comprehensive risk score for effective risk stratification and screening of nasopharyngeal carcinoma. Nat Commun (2021) 12:5189. doi: 10.1038/s41467-021-25402-z 34465768 PMC8408241

[96] Zhang B Choi I-K. Facts and hopes in the relationship of EBV with cancer immunity and immunotherapy. Clin Cancer Res Off J Am Assoc Cancer Res (2022) 28:4363–9. doi: 10.1158/1078-0432.CCR-21-3408 PMC9714122 35686929

[97] Linnerbauer S Behrends U Adhikary D Witter K Bornkamm GW Mautner J. Virus and autoantigen-specific CD4+ T cells are key effectors in a SCID mouse model of EBV-associated post-transplant lymphoproliferative disorders. PloS Pathog (2014) 10:e1004068. doi: 10.1371/journal.ppat.1004068 24853673 PMC4031221

[98] Axelrod ML Cook RS Johnson DB Balko JM. Biological consequences of MHC-II expression by tumor cells in cancer. Clin Cancer Res Off J Am Assoc Cancer Res (2019) 25:2392–402. doi: 10.1158/1078-0432.CCR-18-3200 PMC6467754 30463850

[99] Forero A Li Y Chen D Grizzle WE Updike KL Merz ND. Expression of the MHC class II pathway in triple-negative breast cancer tumor cells is associated with a good prognosis and infiltrating lymphocytes. Cancer Immunol Res (2016) 4:390–9. doi: 10.1158/2326-6066.CIR-15-0243 PMC4878913 26980599

[100] Sconocchia G Eppenberger-Castori S Zlobec I Karamitopoulou E Arriga R Coppola A. HLA class II antigen expression in colorectal carcinoma tumors as a favorable prognostic marker. Neoplasia N Y N (2014) 16:31–42. doi: 10.1593/neo.131568 PMC3924546 24563618

[101] Buetow KH Meador LR Menon H Lu Y-K Brill J Cui H. High GILT expression and an active and intact MHC class II antigen presentation pathway are associated with improved survival in melanoma. J Immunol Baltim Md 1950 (2019) 203:2577–87. doi: 10.4049/jimmunol.1900476 PMC6832889 31591149

[102] Johnson DB Nixon MJ Wang Y Wang DY Castellanos E Estrada MV. Tumor-specific MHC-II expression drives a unique pattern of resistance to immunotherapy via LAG-3/FCRL6 engagement. JCI Insight (2018) 3:e120360, 120360. doi: 10.1172/jci.insight.120360 30568030 PMC6338319

[103] Donia M Andersen R Kjeldsen JW Fagone P Munir S Nicoletti F. Aberrant expression of MHC class II in melanoma attracts inflammatory tumor-specific CD4+ T- cells, which dampen CD8+ T-cell antitumor reactivity. Cancer Res (2015) 75:3747–59. doi: 10.1158/0008-5472.CAN-14-2956 26183926

[104] Chaoul N Tang A Desrues B Oberkampf M Fayolle C Ladant D. Lack of MHC class II molecules favors CD8+ T-cell infiltration into tumors associated with an increased control of tumor growth. Oncoimmunology (2018) 7:e1404213. doi: 10.1080/2162402X.2017.1404213 29399403 PMC5790350

[105] Thome JJC Bickham KL Ohmura Y Kubota M Matsuoka N Gordon C. Early-life compartmentalization of human T cell differentiation and regulatory function in mucosal and lymphoid tissues. Nat Med (2016) 22:72–7. doi: 10.1038/nm.4008 PMC4703455 26657141

[106] Masopust D Vezys V Marzo AL Lefrançois L. Preferential localization of effector memory cells in nonlymphoid tissue. Science (2001) 291:2413–7. doi: 10.1126/science.1058867 11264538

[107] Rosato PC Wijeyesinghe S Stolley JM Nelson CE Davis RL Manlove LS. Virus-specific memory T cells populate tumors and can be repurposed for tumor immunotherapy. Nat Commun (2019) 10:567. doi: 10.1038/s41467-019-08534-1 30718505 PMC6362136

[108] Simoni Y Becht E Fehlings M Loh CY Koo S-L Teng KWW. Bystander CD8+ T cells are abundant and phenotypically distinct in human tumour infiltrates. Nature (2018) 557:575–9. doi: 10.1038/s41586-018-0130-2 29769722

[109] Ning J Gavil NV Wu S Wijeyesinghe S Weyu E Ma J. Functional virus-specific memory T cells survey glioblastoma. Cancer Immunol Immunother (2022) 71:1863–75. doi: 10.1007/s00262-021-03125-w PMC9271132 35001153

[110] Sefrin JP Hillringhaus L Mundigl O Mann K Ziegler-Landesberger D Seul H. Sensitization of tumors for attack by virus-specific CD8+ T-cells through antibody-mediated delivery of immunogenic T-cell epitopes. Front Immunol (2019) 10:1962. doi: 10.3389/fimmu.2019.01962 31555260 PMC6712545

[111] Millar DG Ramjiawan RR Kawaguchi K Gupta N Chen J Zhang S. Antibody-mediated delivery of viral epitopes to tumors harnesses CMV-specific T cells for cancer therapy. Nat Biotechnol (2020) 38:420–5. doi: 10.1038/s41587-019-0404-8 PMC7456461 32042168

[112] Schenkel JM Fraser KA Vezys V Masopust D. Sensing and alarm function of resident memory CD8^+^ T cells. Nat Immunol (2013) 14:509–13. doi: 10.1038/ni.2568 PMC3631432 23542740

[113] Papadopoulos EB Ladanyi M Emanuel D Mackinnon S Boulad F Carabasi MH. Infusions of donor leukocytes to treat Epstein-Barr virus-associated lymphoproliferative disorders after allogeneic bone marrow transplantation. N Engl J Med (1994) 330:1185–91. doi: 10.1056/NEJM199404283301703 8093146

[114] Rooney CM Smith CA Ng CY Loftin S Li C Krance RA. Use of gene-modified virus-specific T lymphocytes to control Epstein-Barr-virus-related lymphoproliferation. Lancet Lond Engl (1995) 345:9–13. doi: 10.1016/s0140-6736(95)91150-2 7799740

[115] Rooney CM Smith CA Ng CY Loftin SK Sixbey JW Gan Y. Infusion of cytotoxic T cells for the prevention and treatment of Epstein-Barr virus-induced lymphoma in allogeneic transplant recipients. Blood (1998) 92:1549–55. doi: 10.1182/blood.V92.5.1549.417k32_1549_1555 9716582

[116] Leen AM Myers GD Sili U Huls MH Weiss H Leung KS. Monoculture-derived T lymphocytes specific for multiple viruses expand and produce clinically relevant effects in immunocompromised individuals. Nat Med (2006) 12:1160–6. doi: 10.1038/nm1475 16998485

[117] McLaughlin LP Rouce R Gottschalk S Torrano V Carrum G Wu M-F. EBV/LMP-specific T cells maintain remissions of T- and B-cell EBV lymphomas after allogeneic bone marrow transplantation. Blood (2018) 132:2351–61. doi: 10.1182/blood-2018-07-863654 PMC6265652 30262660

[118] Leen AM Christin A Myers GD Liu H Cruz CR Hanley PJ. Cytotoxic T lymphocyte therapy with donor T cells prevents and treats adenovirus and Epstein-Barr virus infections after haploidentical and matched unrelated stem cell transplantation. Blood (2009) 114:4283–92. doi: 10.1182/blood-2009-07-232454 PMC2774556 19700662

[119] Khanna R Bell S Sherritt M Galbraith A Burrows SR Rafter L. Activation and adoptive transfer of Epstein-Barr virus-specific cytotoxic T cells in solid organ transplant patients with posttransplant lymphoproliferative disease. Proc Natl Acad Sci U.S.A. (1999) 96:10391–6. doi: 10.1073/pnas.96.18.10391 PMC17898 10468618

[120] Savoldo B Goss JA Hammer MM Zhang L Lopez T Gee AP. Treatment of solid organ transplant recipients with autologous Epstein Barr virus-specific cytotoxic T lymphocytes (CTLs). Blood (2006) 108:2942–9. doi: 10.1182/blood-2006-05-021782 PMC1895521 16835376

[121] Comoli P Labirio M Basso S Baldanti F Grossi P Furione M. Infusion of autologous Epstein-Barr virus (EBV)-specific cytotoxic T cells for prevention of EBV-related lymphoproliferative disorder in solid organ transplant recipients with evidence of active virus replication. Blood (2002) 99:2592–8. doi: 10.1182/blood.v99.7.2592 11895798

[122] Ricciardelli I Blundell MP Brewin J Thrasher A Pule M Amrolia PJ. Towards gene therapy for EBV-associated posttransplant lymphoma with genetically modified EBV-specific cytotoxic T cells. Blood (2014) 124:2514–22. doi: 10.1182/blood-2014-01-553362 PMC4199953 25185261

[123] Brewin J Mancao C Straathof K Karlsson H Samarasinghe S Amrolia PJ. Generation of EBV-specific cytotoxic T cells that are resistant to calcineurin inhibitors for the treatment of posttransplantation lymphoproliferative disease. Blood (2009) 114:4792–803. doi: 10.1182/blood-2009-07-228387 19770360

[124] De Angelis B Dotti G Quintarelli C Huye LE Zhang L Zhang M. Generation of Epstein-Barr virus-specific cytotoxic T lymphocytes resistant to the immunosuppressive drug tacrolimus (FK506). Blood (2009) 114:4784–91. doi: 10.1182/blood-2009-07-230482 PMC2786289 19759356

[125] Comoli P Pedrazzoli P Maccario R Basso S Carminati O Labirio M. Cell therapy of stage IV nasopharyngeal carcinoma with autologous Epstein-Barr virus-targeted cytotoxic T lymphocytes. J Clin Oncol Off J Am Soc Clin Oncol (2005) 23:8942–9. doi: 10.1200/JCO.2005.02.6195 16204009

[126] Kazi S Mathur A Wilkie G Cheal K Battle R McGowan N. Long-term follow up after third-party viral-specific cytotoxic lymphocytes for immunosuppression- and Epstein-Barr virus-associated lymphoproliferative disease. Haematologica (2019) 104:e356–9. doi: 10.3324/haematol.2018.207548 PMC6669158 30792197

[127] Faè DA Martorelli D Mastorci K Muraro E Dal Col J Franchin G. Broadening specificity and enhancing cytotoxicity of adoptive T cells for nasopharyngeal carcinoma immunotherapy. Cancer Immunol Res (2016) 4:431–40. doi: 10.1158/2326-6066.CIR-15-0108 27009165

[128] Zheng Y Parsonage G Zhuang X MaChado LR James CH Salman A. Human leukocyte antigen (HLA) A*1101-restricted epstein-barr virus-specific T-cell receptor gene transfer to target nasopharyngeal carcinoma. Cancer Immunol Res (2015) 3:1138–47. doi: 10.1158/2326-6066.CIR-14-0203-T PMC4456157 25711537

[129] Cho H-I Kim U-H Shin A-R Won J-N Lee H-J Sohn H-J. A novel Epstein-Barr virus-latent membrane protein-1-specific T-cell receptor for TCR gene therapy. Br J Cancer (2018) 118:534–45. doi: 10.1038/bjc.2017.475 PMC5830600 29360818

[130] Zhou J Kang N Cui L Ba D He W. Anti-γδ TCR antibody-expanded γδ T cells: a better choice for the adoptive immunotherapy of lymphoid Malignancies. Cell Mol Immunol (2012) 9:34–44. doi: 10.1038/cmi.2011.16 21666706 PMC4002925

[131] Xiang Z Liu Y Zheng J Liu M Lv A Gao Y. Targeted activation of human Vγ9Vδ2-T cells controls epstein-barr virus-induced B cell lymphoproliferative disease. Cancer Cell (2014) 26:565–76. doi: 10.1016/j.ccr.2014.07.026 25220446

[132] Liu Y Lui KS Ye Z Fung TY Chen L Sit PY. EBV latent membrane protein 1 augments γδ T cell cytotoxicity against nasopharyngeal carcinoma by induction of butyrophilin molecules. Theranostics (2023) 13:458–71. doi: 10.7150/thno.78395 PMC9830437 36632221

[133] Wang X Xiang Z Liu Y Huang C Pei Y Wang X. Exosomes derived from Vδ2-T cells control Epstein-Barr virus-associated tumors and induce T cell antitumor immunity. Sci Transl Med (2020) 12:eaaz3426. doi: 10.1126/scitranslmed.aaz3426 32998970

[134] Wang X Zhang Y Mu X Tu CR Chung Y Tsao SW. Exosomes derived from γδ-T cells synergize with radiotherapy and preserve antitumor activities against nasopharyngeal carcinoma in immunosuppressive microenvironment. J Immunother Cancer (2022) 10:e003832. doi: 10.1136/jitc-2021-003832 35105688 PMC8808451

[135] Younes A Santoro A Shipp M Zinzani PL Timmerman JM Ansell S. Nivolumab for classical Hodgkin’s lymphoma after failure of both autologous stem-cell transplantation and brentuximab vedotin: a multicentre, multicohort, single-arm phase 2 trial. Lancet Oncol (2016) 17:1283–94. doi: 10.1016/S1470-2045(16)30167-X PMC5541855 27451390

[136] Taylor GS Jia H Harrington K Lee LW Turner J Ladell K. A recombinant modified vaccinia ankara vaccine encoding Epstein-Barr Virus (EBV) target antigens: a phase I trial in UK patients with EBV-positive cancer. Clin Cancer Res Off J Am Assoc Cancer Res (2014) 20:5009–22. doi: 10.1158/1078-0432.CCR-14-1122-T PMC4340506 25124688

[137] Orentas RJ Roskopf SJ Nolan GP Nishimura MI. Retroviral transduction of a T cell receptor specific for an Epstein-Barr virus-encoded peptide. Clin Immunol Orlando Fla (2001) 98:220–8. doi: 10.1006/clim.2000.4977 11161978

[138] Kobayashi E Mizukoshi E Kishi H Ozawa T Hamana H Nagai T. A new cloning and expression system yields and validates TCRs from blood lymphocytes of patients with cancer within 10 days. Nat Med (2013) 19:1542–6. doi: 10.1038/nm.3358 24121927

[139] Yang D Shao Q Sun H Mu X Gao Y Jiang R. Evaluation of Epstein-Barr virus latent membrane protein 2 specific T-cell receptors driven by T-cell specific promoters using lentiviral vector. Clin Dev Immunol (2011) 2011:716926. doi: 10.1155/2011/716926 21969838 PMC3182378

[140] Münz C. Redirecting T cells against epstein-barr virus infection and associated oncogenesis. Cells (2020) 9(6):1400. doi: 10.3390/cells9061400 32512847 PMC7349826

[141] Xue S-A Gao L Ahmadi M Ghorashian S Barros RD Pospori C. Human MHC Class I-restricted high avidity CD4+ T cells generated by co-transfer of TCR and CD8 mediate efficient tumor rejection *in vivo* . Oncoimmunology (2013) 2:e22590. doi: 10.4161/onci.22590 23483821 PMC3583927

[142] Chatterjee B Deng Y Holler A Nunez N Azzi T Vanoaica LD. CD8+ T cells retain protective functions despite sustained inhibitory receptor expression during Epstein-Barr virus infection *in vivo* . PloS Pathog (2019) 15:e1007748. doi: 10.1371/journal.ppat.1007748 31145756 PMC6542544

[143] de Vries NL van de Haar J Veninga V Chalabi M Ijsselsteijn ME van der Ploeg M. γδ T cells are effectors of immunotherapy in cancers with HLA class I defects. Nature (2023) 613:743–50. doi: 10.1038/s41586-022-05593-1 PMC9876799 36631610

[144] Rincon-Orozco B Kunzmann V Wrobel P Kabelitz D Steinle A Herrmann T. Activation of V gamma 9V delta 2 T cells by NKG2D. J Immunol Baltim Md 1950 (2005) 175:2144–51. doi: 10.4049/jimmunol.175.4.2144 16081780

[145] Toutirais O Cabillic F Le Friec G Salot S Loyer P Le Gallo M. DNAX accessory molecule-1 (CD226) promotes human hepatocellular carcinoma cell lysis by Vgamma9Vdelta2 T cells. Eur J Immunol (2009) 39:1361–8. doi: 10.1002/eji.200838409 19404979

[146] Wrobel P Shojaei H Schittek B Gieseler F Wollenberg B Kalthoff H. Lysis of a broad range of epithelial tumour cells by human gamma delta T cells: involvement of NKG2D ligands and T-cell receptor- versus NKG2D-dependent recognition. Scand J Immunol (2007) 66:320–8. doi: 10.1111/j.1365-3083.2007.01963.x 17635809

[147] Sell S Dietz M Schneider A Holtappels R Mach M Winkler TH. Control of murine cytomegalovirus infection by γδ T cells. PloS Pathog (2015) 11:e1004481. doi: 10.1371/journal.ppat.1004481 25658831 PMC4450058

[148] Pitard V Roumanes D Lafarge X Couzi L Garrigue I Lafon M-E. Long-term expansion of effector/memory Vdelta2-gammadelta T cells is a specific blood signature of CMV infection. Blood (2008) 112:1317–24. doi: 10.1182/blood-2008-01-136713 PMC2515135 18539896

[149] Khairallah C Déchanet-Merville J Capone M. γδ T cell-mediated immunity to cytomegalovirus infection. Front Immunol (2017) 8:105. doi: 10.3389/fimmu.2017.00105 28232834 PMC5298998

[150] Khairallah C Netzer S Villacreces A Juzan M Rousseau B Dulanto S. γδ T cells confer protection against murine cytomegalovirus (MCMV). PloS Pathog (2015) 11:e1004702. doi: 10.1371/journal.ppat.1004702 25747674 PMC4352080

[151] Zhong H Hu X Janowski AB Storch GA Su L Cao L. Whole transcriptome profiling reveals major cell types in the cellular immune response against acute and chronic active Epstein-Barr virus infection. Sci Rep (2017) 7:17775. doi: 10.1038/s41598-017-18195-z 29259291 PMC5736708

[152] Hassan J Feighery C Bresnihan B Whelan A. Elevated T cell receptor gamma delta + T cells in patients with infectious mononucleosis. Br J Haematol (1991) 77:255–6. doi: 10.1111/j.1365-2141.1991.tb07990.x 1825922

[153] De Paoli P Gennari D Martelli P Cavarzerani V Comoretto R Santini G. Gamma delta T cell receptor-bearing lymphocytes during Epstein-Barr virus infection. J Infect Dis (1990) 161:1013–6. doi: 10.1093/infdis/161.5.1013 2324529

[154] Djaoud Z Guethlein LA Horowitz A Azzi T Nemat-Gorgani N Olive D. Two alternate strategies for innate immunity to Epstein-Barr virus: One using NK cells and the other NK cells and γδ T cells. J Exp Med (2017) 214:1827–41. doi: 10.1084/jem.20161017 PMC5460997 28468758

[155] Djaoud Z Parham P. Dimorphism in the TCRγ-chain repertoire defines 2 types of human immunity to Epstein-Barr virus. Blood Adv (2020) 4:1198–205. doi: 10.1182/bloodadvances.2019001179 PMC7160271 32211881

[156] Zheng BJ Ng SP Chua DTT Sham JST Kwong DLW Lam CK. Peripheral gamma delta T-cell deficit in nasopharyngeal carcinoma. Int J Cancer (2002) 99:213–7. doi: 10.1002/ijc.10326 11979436

[157] Puan KJ Low JSH Tan TWK Wee JTS Tan EH Fong KW. Phenotypic and functional alterations of Vgamma2Vdelta2 T cell subsets in patients with active nasopharyngeal carcinoma. Cancer Immunol Immunother CII (2009) 58:1095–107. doi: 10.1007/s00262-008-0629-8 PMC2695875 19043708

[158] Farnault L Gertner-Dardenne J Gondois-Rey F Michel G Chambost H Hirsch I. Clinical evidence implicating gamma-delta T cells in EBV control following cord blood transplantation. Bone Marrow Transplant (2013) 48:1478–9. doi: 10.1038/bmt.2013.75 23832093

[159] Liu J Gao H Xu L-P Mo X-D Liu R Liang S. Immunosuppressant indulges EBV reactivation and related lymphoproliferative disease by inhibiting Vδ2+ T cells activities after hematopoietic transplantation for blood Malignancies. J Immunother Cancer (2020) 8:e000208. doi: 10.1136/jitc-2019-000208 32221014 PMC7206968

[160] de Witte MA Janssen A Nijssen K Karaiskaki F Swanenberg L van Rhenen A. αβ T-cell graft depletion for allogeneic HSCT in adults with hematological Malignancies. Blood Adv (2021) 5:240–9. doi: 10.1182/bloodadvances.2020002444 PMC7805311 33570642

[161] Maschan M Shelikhova L Ilushina M Kurnikova E Boyakova E Balashov D. TCR-alpha/beta and CD19 depletion and treosulfan-based conditioning regimen in unrelated and haploidentical transplantation in children with acute myeloid leukemia. Bone Marrow Transplant (2016) 51:668–74. doi: 10.1038/bmt.2015.343 26808573

[162] Locatelli F Merli P Pagliara D Li Pira G Falco M Pende D. Outcome of children with acute leukemia given HLA-haploidentical HSCT after αβ T-cell and B-cell depletion. Blood (2017) 130:677–85. doi: 10.1182/blood-2017-04-779769 28588018

[163] Airoldi I Bertaina A Prigione I Zorzoli A Pagliara D Cocco C. γδ T-cell reconstitution after HLA-haploidentical hematopoietic transplantation depleted of TCR-αβ+/CD19+ lymphocytes. Blood (2015) 125:2349–58. doi: 10.1182/blood-2014-09-599423 PMC4440890 25612623

[164] Zvyagin IV Mamedov IZ Tatarinova OV Komech EA Kurnikova EE Boyakova EV. Tracking T-cell immune reconstitution after TCRαβ/CD19-depleted hematopoietic cells transplantation in children. Leukemia (2017) 31:1145–53. doi: 10.1038/leu.2016.321 27811849

[165] Fujishima N Hirokawa M Fujishima M Yamashita J Saitoh H Ichikawa Y. Skewed T cell receptor repertoire of Vdelta1(+) gammadelta T lymphocytes after human allogeneic haematopoietic stem cell transplantation and the potential role for Epstein-Barr virus-infected B cells in clonal restriction. Clin Exp Immunol (2007) 149:70–9. doi: 10.1111/j.1365-2249.2007.03388.x PMC1942033 17425654

[166] Xu Y Xiang Z Alnaggar M Kouakanou L Li J He J. Allogeneic Vγ9Vδ2 T-cell immunotherapy exhibits promising clinical safety and prolongs the survival of patients with late-stage lung or liver cancer. Cell Mol Immunol (2021) 18:427–39. doi: 10.1038/s41423-020-0515-7 PMC8027668 32939032

[167] Di Lorenzo B Simões AE Caiado F Tieppo P Correia DV Carvalho T. Broad cytotoxic targeting of acute myeloid leukemia by polyclonal delta one T cells. Cancer Immunol Res (2019) 7:552–8. doi: 10.1158/2326-6066.CIR-18-0647 30894378

[168] Lamb LS Pereboeva L Youngblood S Gillespie GY Nabors LB Markert JM. A combined treatment regimen of MGMT-modified γδ T cells and temozolomide chemotherapy is effective against primary high grade gliomas. Sci Rep (2021) 11:21133. doi: 10.1038/s41598-021-00536-8 34702850 PMC8548550

